# The meta and bioinformatics analysis of GRP78 expression in gastric cancer

**DOI:** 10.18632/oncotarget.20318

**Published:** 2017-08-18

**Authors:** Hua-Chuan Zheng, Bao-Cheng Gong, Shuang Zhao

**Affiliations:** ^1^ Department of Experimental Oncology and Animal Center, Shengjing Hospital of China Medical University, Shenyang 110004, China

**Keywords:** GRP78, gastric cancer, meta analysis, bioinformatics analysis

## Abstract

GRP78 is a molecular chaperone located in endoplasmic reticulum, and induces folding and assembly of newly-synthesized proteins, proteasome degradation of aberrant proteins, and translocation of secretory proteins, autophagy, and epithelial-mesenchymal transition. We performed a systematic meta- and bioinformatics analysis through multiple online databases up to March 14, 2017. It was found that up-regulated GRP78 expression in gastric cancer, compared with normal mucosa at both protein and mRNA levels (*p* < 0.05). GRP78 expression was positively correlated with depth of invasion, TNM staging and dedifferentiation of gastric cancer (*p* < 0.05), while its mRNA expression was negatively correlated with depth of invasion, histological grading and dedifferentiation (*p* < 0.05). A positive association between GRP78 expression and unfavorable overall survival was found in patients with gastric cancer (*p* < 0.005). A higher *GRP78* mRNA expression was positively correlated with overall and progression-free survival rates of all cancer patients, even stratified by aggressive parameters, or as an independent factor (*p* < 0.05). These findings indicated that GRP78 expression might be employed as a potential marker to indicate gastric carcinogenesis and subsequent progression, even prognosis.

## INTRODUCTION

Endoplasmic reticulum (ER) stress is initiated by oxidative stress, glucose deprivation, chemical toxicity, alterations in intracellular Ca^2+^ levels, and blockade of glycosylation and hypoxia, and subsequently activates the expression of glucose-related proteins (GRPs) and CHOP by ER stress response element. It results in cell survival via translational attenuation to limit further accumulation of misfolded proteins, but prolonged or strong ER does apoptosis. GRP78 is a 78 kDa molecular chaperone located in ER lumen, and is also named as Binding immunoglobulin protein (BiP) or heat shock protein 5 (HSPA5). It is responsible for folding and assembly of newly- synthesized proteins, transport retrogradation across the ER membrane, proteasome degradation of aberrant proteins, and translocation of secretory proteins [1, 2].

GRP78 overexpression promotes autophagy, evidenced by increased VPS34 and LC3-II, and decreased p62 and LC3-I [3]. It activates PI3K-mediated autophagy pathway and induces autophagic degradation of IKKβ, which causes inactivation of NF-κB pathway, and subsequently alters the expression of PKM2 and HIF-1α [4]. GRP78 confers drug resistance by aggresome delivery to autophagosomes [5] or c-src/LSF/TS activation [6] respectively. Zhang et al. [7] found that cancer cells promoted cell surface relocalization of GRP78 which interacted with PI3K and enhanced PIP3 production. Cell-surface GRP78 might facilitate the migration and invasion of colorectal cancer cells by regulating cell-matrix adhesion and the degradation of extracellular matrix, which was partly mediated through uPA-uPAR protease system [8]. Blockade of cripto binding to cell surface GRP78 inhibited oncogenic cripto signaling via MAPK/PI3K and Smad2/3 pathways [9]. Cell surface GRP78 directly bound to and phosphorylated c-src for EGFR activation, promoting the invasion and metastasis of hepatocellular carcinoma (HCC) cells [10], and accelerating the proliferation and migration of breast cancer cells by activating STAT3 [11]. Li et al. [12] found that GRP78 was secreted from colon cancer cells via exosomes. Monoclonal antibody against cell surface GRP78 suppressed tumor growth and metastasis by weakening PI3K/Akt signaling [13]. Histone deacetylase inhibitors blocked GRP78 release by inducing its aggregation in ER [12]. Reportedly, secretory GRP78 facilitated cell proliferation of colon cancer cells via PI3K/Akt signaling [14], and stimulated the differentiation of bone marrow mesenchymal stem cells to cancer-associated fibroblasts by activating TGF-β/Smad signaling pathway [15].

Body weight, organ development and integrity were not impaired, and cancer incidence and inflammation were not affected in GRP78 (+/−) mice [16]. At 3 months, PTEN (f/f)GRP78(f/f) livers showed hepatomegaly, activation of lipogenic genes, exacerbated steatosis and liver injury, whereas HCC was developed at 8–9 months [17]. Dong et al. [18] found that GRP78 heterozygosity impeded cancer growth by suppressing tumor cell proliferation and promoting apoptosis, and exhibited dramatic reduction in the microvessel density of the endogenous mammary tumors. Conditional heterozygous knockout of GRP78 in the host endothelial cells showed severe reduction of tumor angiogenesis and metastatic growth with minimal effect on microvessel density of normal tissue. In gastric cancer cells, GRP78 knockdown inhibited proliferation and enhanced apoptosis, whereas MEK inhibition blocked GRP78 up-regulation and had the same effects [19–20]. Gastric adenocarcinoma patients with the combined GRP78 rs391957 C/T and T/T genotype were at higher risk for tumor recurrence and death than those with C/C [21]. Our previous study also showed that GRP78 expression was upregulated in gastric cancer, and positively correlated with tumor size, depth of invasion, lymphatic and venous invasion, lymph node metastasis, TNM staging, and poor prognosis of gastric cancer [22]. In the present study, we performed a meta- and bioinformatics analysis to clarify the clinicopathological and prognostic significances of GRP78 expression at both mRNA and protein levels.

## RESULTS

### Characteristics of eligible studies

**Figure 1:**
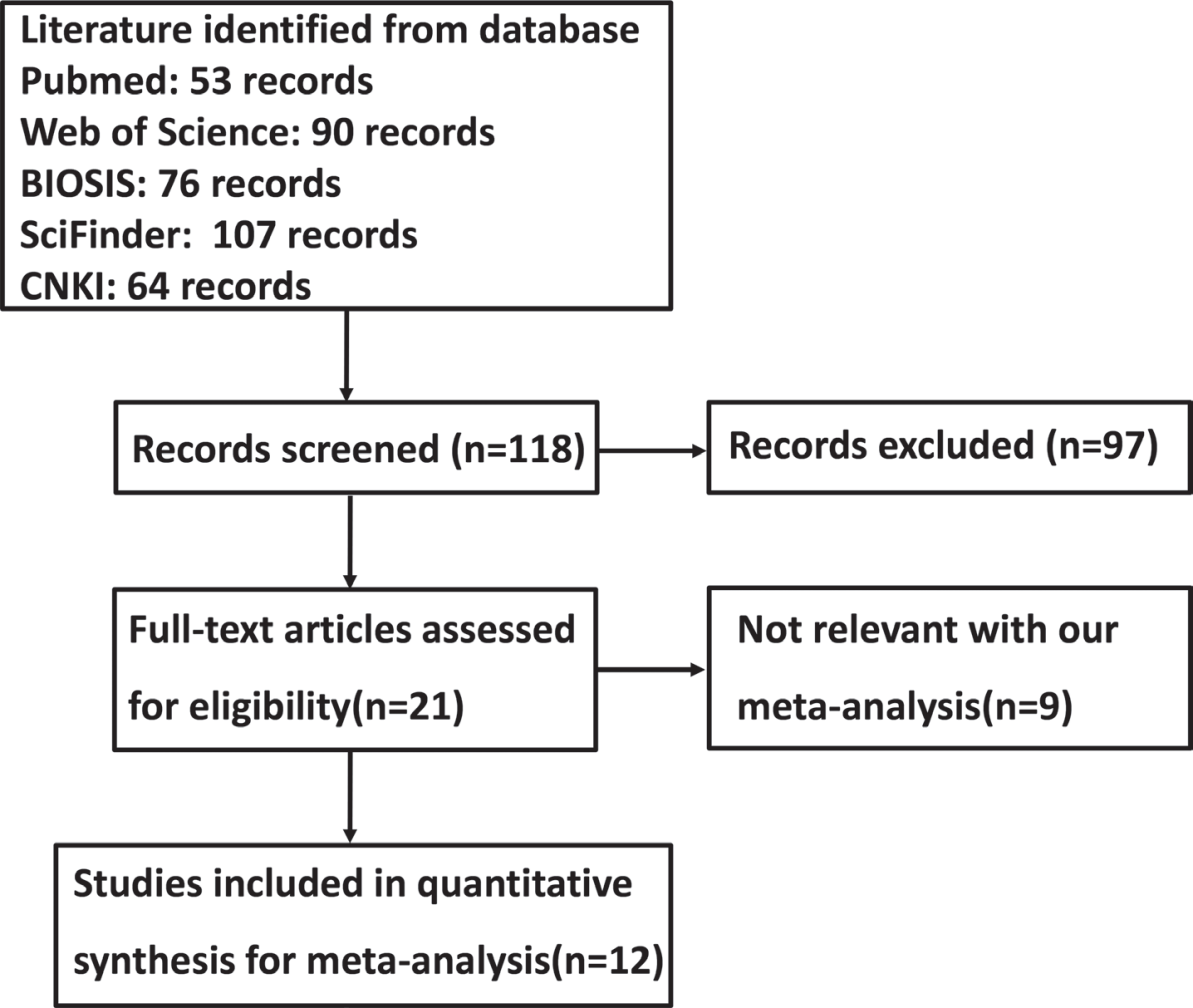
Flow diagram of the selection process in this meta-analysis

Figure 1 is the flow diagram of paper selection in our meta-analysis. As shown in Table 1, a total of 12 articles on the relationship between GRP78 expression and cancer risk, clinicopathological or prognostic parameters of gastric cancer were retrieved for our meta-analysis by immunohistochemistry in PubMed, Web of Science, BIOSIS, SciFinder and CNKI. Only 3 articles contained the samples of normal gastric mucosa [22–24]. There appeared the comparison between GRP78 expression and clinicopathological characteristics of gastric cancer in 12 pieces of paper, including sex, depth of invasion, lymph node metastasis, TNM staging and Lauren's classification [22–33]. Finally, the authors discussed the prognostic significance of GRP78 expression in 6 articles [22, 24, 25, 28, 30, 33].

**Table 1:** Main characteristics of eligible studies

First author	Year	Country	Ethnicity	Antibody source	Cases	Control	Risk to cancer	Outcome	Quality
Zhang J	2006	China	Asian	Santa Cruz	86			Negative	8
Zheng HC	2008	Japan	Asian	Santa Cruz	487	83	Up	Negative	9
Yang L	2014	China	Asian	Zhongshan	237			Negative	8
Zhang XC	2005	China	Asian	Santa Cruz					8
Xu FY	2010	China	Asian	Santa Cruz	65				8
Peng CL	2013	China	Asian	Ruiqi	60	60	Up	Negative	8
Fu ZQ	2014	China	Asian	Abcam	34				8
Wei H	2014	China	Asian	Santa Cruz	80			Negative	8
Li LN	2014	China	Asian	Proteintech	60	20	Up		8
He ZX	2015	China	Asian	Boster	48				8
Hu YW	2016	China	Asian	Santa Cruz	90				8
Ge JJ	2016	China	Asian	Abcam	172				8

### Association between GRP78 expression and cancer susceptibility of gastric mucosa

**Figure 2:**
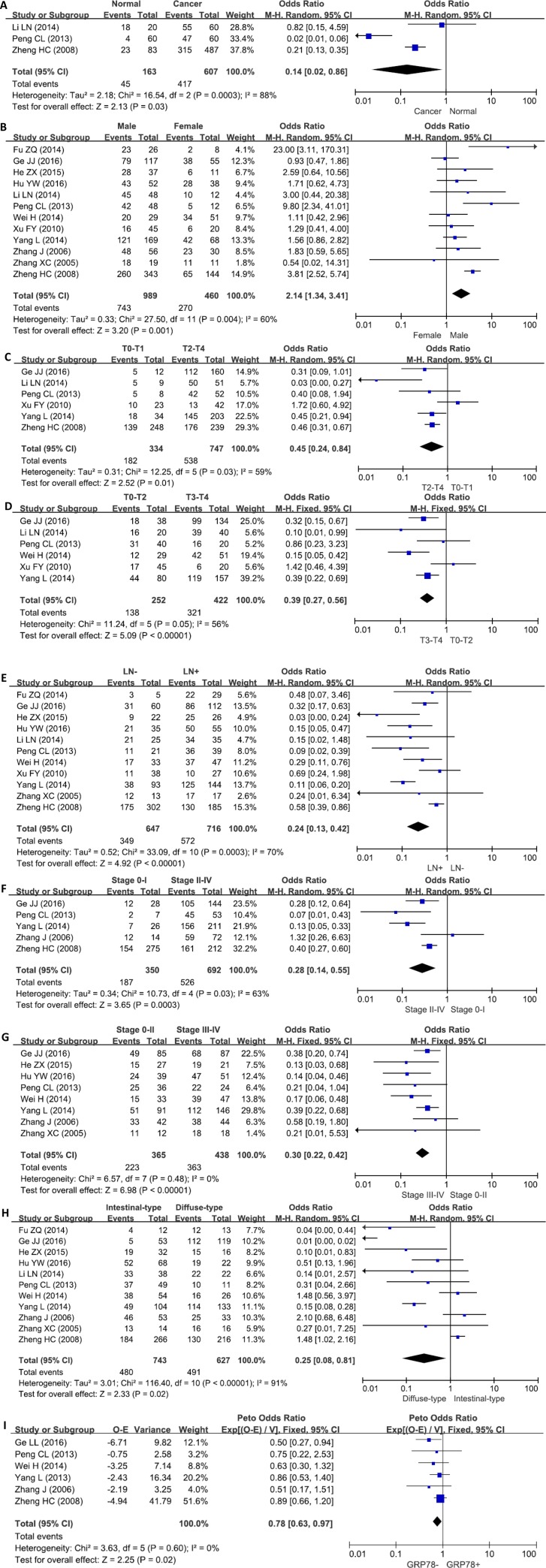
Forest plot for the relationship between GRP78 expression and clinicopathological parameters of gastric cancer (**A**) gastric carcinogenesis (cancer *vs* normal mucosa); (**B**) correlation between sex and GRP78 expression (male *vs* female); (**C**) correlation between depth of invasion and GRP78 expression (T0–1 *vs* T2–4); (**D**) correlation between depth of invasion and GRP78 expression (T0–2 *vs* T3–4); (**E**) correlation between lymph node metastasis (LN) and GRP78 expression (LN− *vs* LN+); (**F**) correlation between TNM staging and GRP78 expression (stage 0–I *vs* stage II–IV); (**G**) correlation between TNM staging and GRP78 expression (stage 0–II *vs* stage III–IV); (**H**) correlation between differentiation and GRP78 expression (intestinal-type *vs* diffuse-type); (**I**) correlation between survival and GRP78 expression (GRP78+ *vs* GRP78−).

We analyzed the association between GRP78 expression and cancer susceptibility of gastric normal mucosa in 3 studies with 607 cancers and 163 controls. As a result, we found up-regulated GRP78 expression in gastric cancer, compared with normal mucosa (Figure 2A, *p* = 0.03).

### Association between GRP78 expression and clinicopathological parameters of gastric cancer

As shown in Figure 2B, the male patients with gastric cancer showed a higher GRP78 expression than female ones (*p* < 0.05). A higher GRP78 expression was detected in T2–4 than T0–1 gastric cancers (Figure 2C, *p* = 0.01), and in T3–4 than T0–2 cancers (Figure 2D, *p* < 0.00001). GRP78 expression was positively related to lymph node metastasis of gastric cancer (Figure 2E, *p* < 0.00001). Gastric cancers with stage II–IV showed GRP78 overexpression, compared with ones with stage 0-I (Figure 2F, *p* < 0.00001). The stage III–IV cancers displayed a higher expression of GRP78 than stage 0–II ones (Figure 2G, *p* = 0.00003). GRP78 protein was less expressed in intestinal-type than diffuse-type carcinoma (Figure 2H, *p* = 0.02).

### Association between GRP78 expression and survival rate of gastric cancer

As indicated in Figure 2I, the pooled result from 6 datasets demonstrated a negative association between GRP78 expression and unfavorable overall survival in patients with gastric cancer (HR = 0.78, 95% CI: 0.63–0.97, *p* = 0.02).

### Publication bias

**Figure 3:**
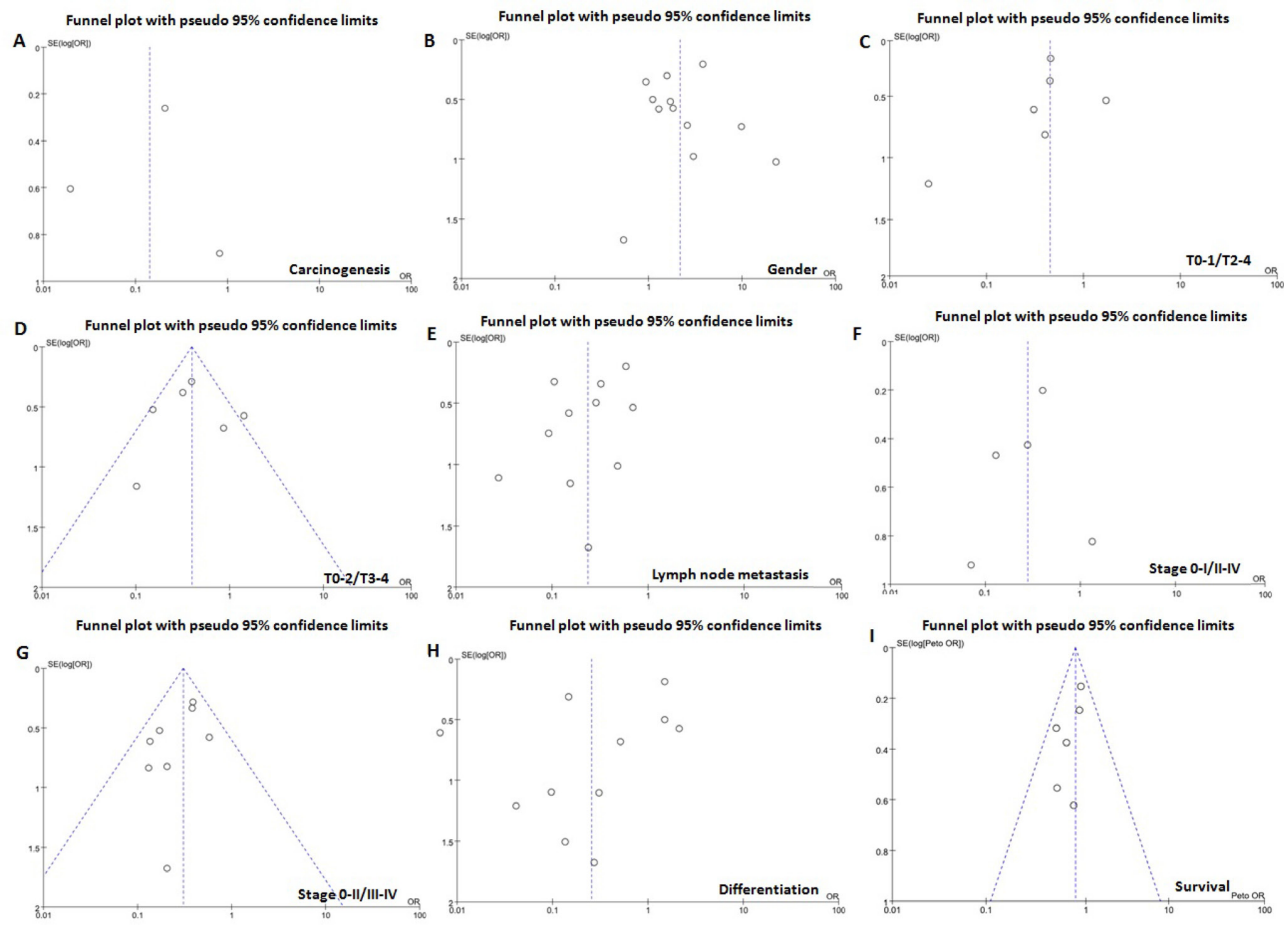
Funnel plot for publication bias test between GRP78 expression and gastric carcinogenesis or progression The bias was analyzed about risk degrees of GRP78 expression in gastric mucosa (**A**) afor gastric carcinogenesis. Additionally, it was tested between GRP78 expression and clinicopathological features of gastric cancer, including age (**B**), depth of invasion (**C** and **D**), lymph node metastasis (**E**), TNM staging (**F** and **G**), and differentiation (**H**) and prognosis (**I**).

The heterogeneity test was performed as shown in Figure 3. Sensitivity analysis was used to evaluate individual study's influence on the pooled results by deleting one single study each time from pooled analysis. As a result, the carcinogenesis risk of GRP78-positive mucosa from Li's study had a significant effect on the pooled OR. When this study was excluded, the heterogeneity test was significantly reduced (data not shown).

### The clinicopathological and prognostic significances of *GRP78* mRNA expression in gastric cancers

**Figure 4:**
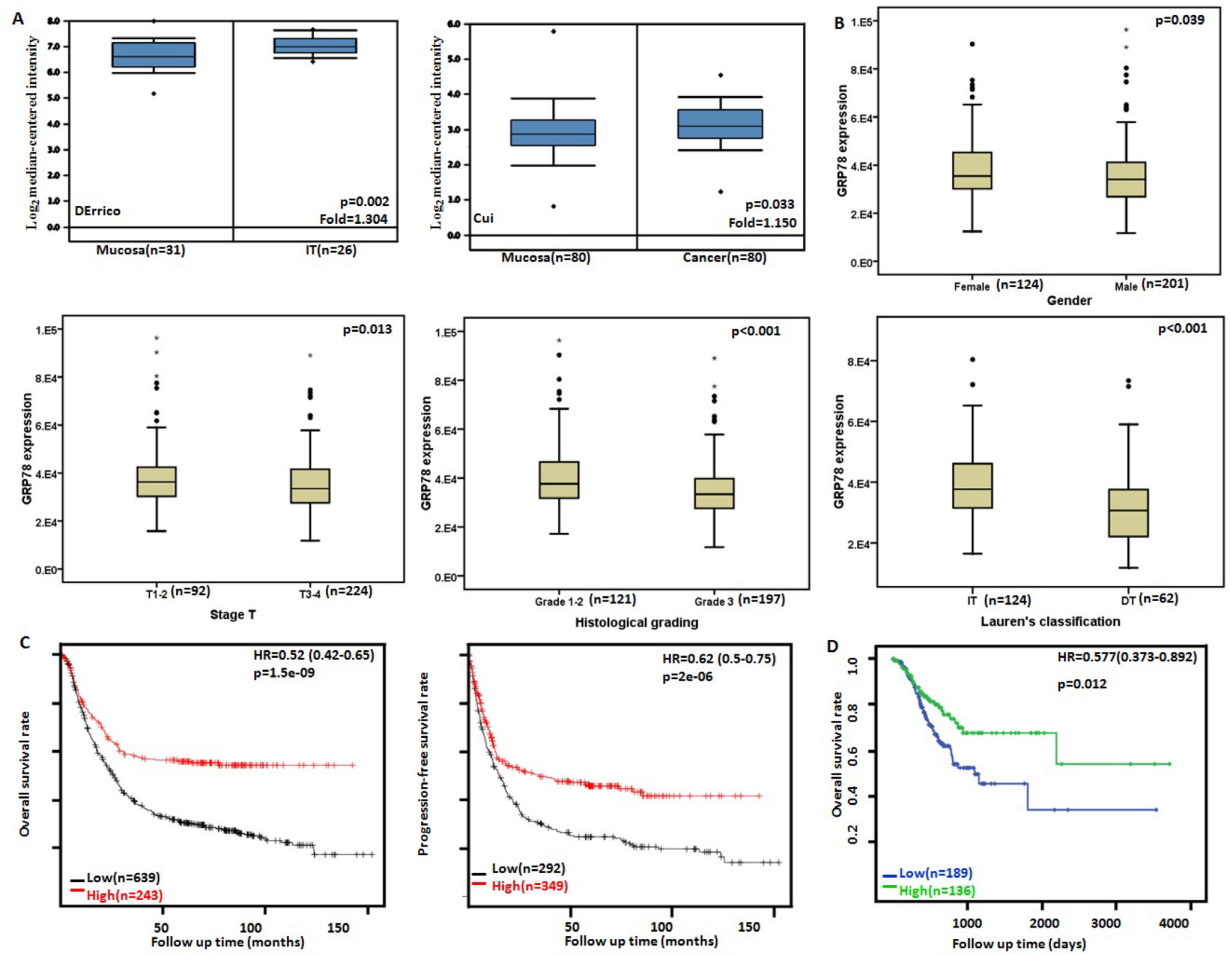
*GRP78* mRNA expression in gastric carcinogenesis and subsequent progression Cui's and DErrico's datasets were employed for bioinformatics analysis to analyze *GRP78* mRNA expression during gastric carcinogenesis. A higher *GRP78* mRNA expression was detectable in gastric cancer than that in normal gastric mucosa (**A**) *p* < 0.05, even in intestinal-type (IT) carcinoma. TCGA database shows that *GRP78* mRNA was more expressed in female than male gastric cancers (**B**) *p* < 0.05. *GRP78* mRNA expression was negatively correlated with T staging, histological grading and differentiation of gastric cancers (B) *p* < 0.05. According to the data from KM plotter, *GRP78* mRNA expression was positively related to both overall and progression-free survival rates of the patients with gastric cancer (**C**). According to TGCA database, *GRP78* mRNA expression was positively linked to overall survival rate of the patients with gastric cancer (**D**). HR, hazard ratio.

Then, we used DErrico's and Cui's datasets to perform bioinformatics analysis and found that *GRP78* mRNA expression was higher in gastric cancer than normal tissues, even in intestinal-type carcinoma (Figure 4A, *p* < 0.05). In TCGA data, *GRP78* mRNA expression was higher in female with male gastric cancers (Figure 4B, *p* < 0.001). It was negatively correlated with depth of invasion and histological grading of gastric cancer as well (Figure 4B, *p* < 0.05). A higher *GRP78* mRNA expression was detectable in intestinal-type carcinoma than diffuse-type counterpart (Figure 4B, *p* < 0.001).

According to Kaplan-Meier plotter, we found that a higher *GRP78* mRNA expression was positively correlated with overall and progression-free survival rates of all cancer patients (Figure 4C, *p* < 0.05). As summarized in Table 2, the cancer patients with high *GRP78* mRNA expression showed a long overall survival time than those with its low expression, even stratified by gender, treatment, Lauren's classification and Her2 immunoreactivity (*p* < 0.05). The overall or progression-free survival rate of the patient with T2–T4, lymph-node-positive, and M0 cancers was higher in the group of high *GRP78* mRNA expression than that of its low expression (*p* < 0.05).

**Table 2:** The prognostic significance of *GRP78* mRNA in gastric cancer

Clinicopathological features	Overall survival	*p*	Progression-free survival	*p*
	Hazard ratio		Hazard ratio	
Sex				
Female	0.48 (0.32−0.7)	9.1e−05	0.53 (0.36−0.79)	0.0013
Male	0.55 (0.42−0.72)	7.4e−06	0.58 (0.44−0.76)	5.7e−05
T				
2	0.42 (0.27−0.65)	7.1e−05	0.49 (0.32−0.75)	0.00077
3	0.58 (0.38−0.89)	0.011	0.69 (0.46−1.02)	0.063
4	0.36 (0.14−0.91)	0.025	0.51 (0.22−1.17)	0.11
N				
0	0.54 (0.22−1.35)	0.18	0.57 (0.21−1.58)	0.28
1–3	0.45 (0.34−0.6)	3e−08	0.52 (0.4−0.69)	2.2e−06
1	0.49 (0.32−0.74)	0.00055	0.53 (0.36−0.78)	0.0012
2	0.32 (0.18−0.59)	0.00011	0.39 (0.23−0.67)	0.00042
3	0.28 (0.14−0.56)	1e−04	0.38 (0.2−0.73)	0.0024
M				
0	0.45 (0.33−0.61)	1.2e−07	0.53 (0.4−0.7)	9.5e−06
1	0.59 (0.3−1.17)	0.12	1.89 (0.95−3.76)	0.066
TNM staging				
I	0.46 (0.14− 1.46)	0.18	0.65 (0.19−2.18)	0.48
II	0.24 (0.09−0.61)	0.0012	0.31 (0.13−0.73)	0.0045
III	0.57 (0.41−0.79)	0.00068	0.55 (0.35−0.87)	0.0099
IV	0.49 (0.31−0.76)	0.0013	0.63 (0.41−0.96)	0.032
Differentiation				
Well-differentiated	-	-	-	-
Moderately-differentiated	0.68 (0.35−1.32)	0.25	0.69 (0.36−1.3)	0.24
Poorly-differentiated	1.24 (0.83−1.87)	0.29	1.22 (0.77−1.92)	0.4
Lauren's classification				
Intestinal-type	0.44 (0.3−0.62)	3e−06	0.57 (0.39−0.82)	0.002
Diffuse-type	0.5 (0.34−0.72)	0.00017	0.52 (0.36−0.76)	5e−04
Her2 positivity				
−	0.49 (0.37−0.65)	3.3e−07	0.56 (0.43−0.75)	4.4e−05
+	0.7 (0.52−0.93)	0.014	0.68 (0.49−0.93)	0.016
Perforation				
−	0.79 (0.5−1.23)	0.29	0.8 (0.52−1.25)	0.33
Treatment				
Surgery alone	0.55 (0.4−0.76)	2e−04	0.67 (0.5−0.9)	0.0073
Other adjuvant	0.07 (0.01−0.5)	0.00047	0.28 (0.12−0.63)	0.0011

According to TCGA's database, univariate analysis showed a positive link between *GRP78* mRNA expression and the overall better prognosis of the patients with gastric cancer (Figure 4C, *p* < 0.05). Multivariate analysis using Cox's hazard proportional model indicated that *GRP78* mRNA expression was independent prognostic factor for gastric cancer (Table 3, *p* < 0.05).

**Table 3:** Multivariate analysis of hazard factors of the prognosis of the patients with gastric cancer

Clinicopathological features	Hazard ratio (95% CI)	*p*
Gender (female/ male)	0.786 (0.515–1.199)	0.264
Stage T (T1–2/T3–4)	1.006 (0.577–1.756)	0.983
Lymph node status (−/+)	0.999 (0.557–1.792)	0.997
Distant metastasis (−/+)	1.605 (0.557–4.619)	0.381
TNM staging (I–II/III–IV)	0.741 (0.399–1.375)	0.342
Histological staging(G1–2/3)	1.121 (0.879–1.429)	0.357
*GRP78* mRNA expression (low/ high)	0.572 (0.357–0.916)	0.020

## DISCUSSION

GRP78 silencing was reported to increase essential polyunsaturated fat accumulation of breast cancer cells, due to an inhibition of mitochondrial fatty acid transport and a reduction of fatty acid oxidation [34]. In pancreatic ductal cancer cells, GRP78 enhanced the proliferation, migration and invasion by up-regulating the expression of CyclinD1, CDK 4, CDK6, p-STAT3, JAK2, RhoA, ROCK1, and Smad4 [35]. GRP78 promoted cancer metastasis via up-regulation of MMP-2 and MMP-9, the induction of epithelial-mesenchymal transition or activation of NRF-2/HO-1 pathway [36–38]. Xie et al. [39] reported that GRP78 inhibited apoptosis and attenuated gemcitabine chemosensitivity in breast cancer cell via Akt/ mitochondrial apoptotic pathway. Li et al. [40] found that GRP78 mediated radiotherapeutic resistance of a stem cell-like subpopulation within breast cancer cells. To investigate the clinicopathological and prognostic significances of GRP78 expression, we analyzed 12 studies, which met specific inclusion criteria and had moderate to high quality according to their NOS scores.

Ciortea et al. [41] found that plasma GRP78 level was significantly higher in patients with endometrial cancer than the control group. Android fat distribution had a positive correlation with plasma GRP78 level, but versa for gynoid fat. Raiter et al. [42] demonstrated that anti-GRP78 titer in patients with colorectal polyp or cancer was higher than that in healthy subjects. Consistent with the data about esophageal cancer, medullary thyroid carcinoma, pancreatic ductal adenocarcinoma, colorectal cancer, renal clear cell carcinoma, astrocytoma, prostate cancer, lung cancer and hepatocellular carcinoma [35, 43–50], we found up-regulated GRP78 expression in gastric cancer, compared with gastric mucosa at both mRNA and protein levels, suggesting that GRP78 hyperexpression was positively linked to gastric carcinogenesis. Although anti-GRP78 antibodies come from 6 companies, and different statistical methods are employed, GRP78 expression and its correlation with clinicopathological parameters are comparatively consistent, indicating that these antibodies mainly recognizes GRP78 protein and its expression trend is not determined by statistics. Here, there appeared a positive correlation between GRP78 protein expression and aggressiveness, such as depth of invasion, lymph node metastasis and TNM staging, but the converse was true for *GRP78* mRNA expression, indicating that aberrant GRP78 expression might be employed to indicate the pathological behaviors of gastric cancer. This is not surprising since mRNA levels from an expressed gene do not usually predict the corresponding protein levels because it takes a long distance from mRNA to protein by translation and posttranslational modification.

Reportedly, GRP78 expression was positively related to the poor prognosis of the patients with pancreatic and rectal cancers [35, 51]. It might be also demonstrated to indicate the worse prognosis of esophageal cancer, melanoma, advanced laryngeal squamous cell carcinoma, tongue cancer, and glioblastoma as an independent factor [43, 47, 52–54]. Ma et al. [55] reported that the overall survival of patients with high serum GRP78 level was significantly poorer than those with low GRP78 level. Our meta-analysis showed that GRP78 expression was positively linked to the worse prognosis of the patients with gastric cancer. However, our bioinformatics data indicated that *GRP78* mRNA expression was positively associated with overall and progression-free survival rates of the patient with gastric cancer, even stratified by clinicopathological features or as an independent factor. The paradoxical findings might be attributable to their correlation with aggressive behaviors of gastric cancer.

In conclusion, GRP78 expression underwent an up-regulation from gastric carcinogenesis at both protein and mRNA levels. GPR78 protein expression was positively correlated with depth of invasion, lymph node metastasis, TNM staging and dedifferentiation of gastric cancer, but versa for *GRP78* mRNA. GRP78 protein expression might be employed as a good potential marker for worse prognosis of gastric cancer patients. *GRP78* mRNA expression has the opposite results, even stratified by aggressive features or as an independent factor. Several limitations should be noted in our meta-analysis. Firstly, the potential publication bias stems from published results being predominantly positive. Secondly, patient populations in our study were limited, as patients came only from Asia. Thirdly, all of the survival data were extracted from survival curves, which may introduce subjective bias. Fourthly, this small sample size limits the power to detect the associations in some articles.

## MATERIALS AND METHODS

### Identification of eligible studies and data extraction

We performed a publication search using PubMed, Web of Science, BIOSIS and SciFinder updated on March 14, 2017. The following search terms were used: (GRP78 OR Bip OR HSPA5) AND (gastric OR stomach) AND (cancer OR carcinoma OR adenocarcinoma). Searching was done without restriction on language or publication years. Inclusion criteria for studies: (1) articles to observe the alteration in GRP78 expression in gastric cancer by immunohistochemistry; (2) papers to compare GRP78 expression with pathobiological behaviors and prognosis of gastric cancer by immunohistochemistry. Exclusion criteria included: (1) abstract, comment, review and meeting; (2) duplication of the previous publications; (3) Western blot, RT-PCR, cDNA microarray, or transcriptomic sequencing for GRP78 expression; (4) lack of sufficient information.

### Data extraction

Based on the inclusion criteria, two reviewers (HC Zheng and BC Gong) independently extracted information from all eligible publications. The following information was included in each study: name of first author, year of publication, country, ethnicity, antibody company, numbers of cases and controls, expression alteration, and follow-up outcome. Regarding survival analysis, we used Engauge Digitizer software to extract data from Kaplan-Meier curves and calculated the Hazard ratios (HR) and their corresponding 95% confidence intervals (CI). Any disagreement was resolved through discussion until the two reviewers reached a consensus.

### Quality score assessment

Two reviewers (HC Zheng and BC Gong) independently assessed the quality of the included studies according to Newcastle Ottawa Scale (NOS) (http://www.ohri.ca/programs/clinical_epidemiology/oxford.htm). The scale consists of three components related to sample selection, comparability and ascertainment of outcome.

### Bioinformatics analysis

The individual gene expression level of *GRP78* was analyzed using Oncomine (www.oncomine.org), a cancer microarray database and web-based data mining platform for a new discovery from genome-wide expression analyses. We compared the differences in *GRP78* mRNA level between gastric normal tissue and cancer. All data were log-transformed, median centered per array, and standard deviation normalized to one per array. The expression (RNA-seqV2) and clinicopathological data of 392 gastric cancer patients were downloaded from the Cancer Genome Atlas (TCGA) database by TCGA- assembler in R software. We integrated the raw data, analyzed *GRP78* mRNA expression in gastric cancer, and compared it with clinicopathological and prognostic data of the patients with gastric cancer. Additionally, the prognostic significance of *GRP78* mRNA was also analyzed using Kaplan-Meier plotter (http://kmplot.com).

### Statistic analysis

HWE was evaluated using Chi-square test in control groups of each study. Strength of association between GRP78 expression and cancer risk was assessed by odds ratios with 95% confidence intervals. Statistical significance of the pooled OR was determined by *Z* test. If there was no significant heterogeneity, the fixed effect model (Mantel-Haenszel method) would be employed. Otherwise, the random effect model (DerSimonian and Laird method) would be used excluding prognostic analysis. Heterogeneity effect was then quantified by *I*^2^ test, which was subdivided into low, moderate and high degrees of heterogeneity according to the cut-off values of 25%, 50% and 75% respectively. Publication bias was evaluated by funnel plot and quantified by Begg's test and Egger's test to assess funnel plot asymmetry. Meta-analyses were performed with Revman software 5.3 and data from TCGA database was dealt with SPSS 10.0 software using student *t* test. *Kaplan-Meier* survival plots were generated and comparisons between survival curves were made with the log-rank statistic. Cox's proportional hazards model was employed for multivariate analysis. Two-sided *p* < 0.05 was considered as statistically significant.

## References

[1] Zhang LH, Zhang X (2010). Roles of GRP78 in physiology and cancer. J Cell Biochem.

[2] Roller C, Maddalo D (2013). The Molecular Chaperone GRP78/BiP in the development of chemoresistance: mechanism and possible treatment. Front Pharmacol.

[3] Wang Y, Wu H, Li Z, Yang P, Li Z (2017). A positive feedback loop between GRP78 and VPS34 is critical for GRP78-mediated autophagy in cancer cells. Exp Cell Res.

[4] Li Z, Wang Y, Newton IP, Zhang L, Ji P, Li Z (2015). GRP78 is implicated in the modulation of tumor aerobic glycolysis by promoting autophagic degradation of IKKβ. Cell Signal.

[5] Abdel Malek MA, Jagannathan S, Malek E, Sayed DM, Elgammal SA, Abd El-Azeem HG, Thabet NM, Driscoll JJ (2015). Molecular chaperone GRP78 enhances aggresome delivery to autophagosomes to promote drug resistance in multiple myeloma. Oncotarget.

[6] Gu YJ, Li HD, Zhao L, Zhao S, He WB, Rui L Su C, Zheng HC, Su RJ (2015). GRP78 confers the resistance to 5-FU by activating the c-Src/LSF/TS axis in hepatocellular carcinoma. Oncotarget.

[7] Zhang Y, Tseng CC, Tsai YL, Fu X, Schiff R, Lee AS (2013). Cancer cells resistant to therapy promote cell surface relocalization of GRP78 which complexes with PI3K and enhances PI(3,4,5)P3 production. PLoS One.

[8] Li Z, Zhang L, Zhao Y, Li H, Xiao H, Fu R, Zhao C, Wu H, Li Z (2013). Cell-surface GRP78 facilitates colorectal cancer cell migration and invasion. Int J Biochem Cell Biol.

[9] Kelber JA, Panopoulos AD, Shani G, Booker EC, Belmonte JC, Vale WW, Gray PC (2009). Blockade of Cripto binding to cell surface GRP78 inhibits oncogenic Cripto signaling via MAPK/PI3K and Smad2/3 pathways. Oncogene.

[10] Zhao S, Li H, Wang Q, Su C, Wang G, Song H, Zhao L, Luan Z, Su R (2015). The role of c-Src in the invasion and metastasis of hepatocellular carcinoma cells induced by association of cell surface GRP78 with activated α2M. BMC Cancer.

[11] Yao X, Liu H, Zhang X, Zhang L, Li X, Wang C, Sun S (2015). Cell surface GRP78 accelerated breast cancer cell proliferation and migration by activating STAT3. PLoS One.

[12] Li Z, Zhuang M, Zhang L, Zheng X, Yang P, Li Z (2016). Acetylation modification regulates GRP78 secretion in colon cancer cells. Sci Rep.

[13] Liu R, Li X, Gao W, Zhou Y, Wey S, Mitra SK, Krasnoperov V, Dong D, Liu S, Li D, Zhu G, Louie S, Conti PS (2013). Monoclonal antibody against cell surface GRP78 as a novel agent in suppressing PI3K/AKT signaling, tumor growth, and metastasis. Clin Cancer Res.

[14] Fu R, Yang P, Wu HL, Li ZW, Li ZY (2014). GRP78 secreted by colon cancer cells facilitates cell proliferation via PI3K/Akt signaling. Asian Pac J Cancer Prev.

[15] Peng Y, Li Z, Li Z (2013). GRP78 secreted by tumor cells stimulates differentiation of bone marrow mesenchymal stem cells to cancer-associated fibroblasts. Biochem Biophys Res Commun.

[16] Lee AS, Brandhorst S, Rangel DF, Navarrete G, Cohen P, Longo VD, Chen J, Groshen S, Morgan TE, Dubeau L (2017). Effects of prolonged GRP78 haploinsufficiency on organ homeostasis, behavior, cancer and chemotoxic resistance in aged mice. Sci Rep.

[17] Chen WT, Zhu G, Pfaffenbach K, Kanel G, Stiles B, Lee AS (2014). GRP78 as a regulator of liver steatosis and cancer progression mediated by loss of the tumor suppressor PTEN. Oncogene.

[18] Dong D, Stapleton C, Luo B, Xiong S, Ye W, Zhang Y, Jhaveri N, Zhu G, Ye R, Liu Z, Bruhn KW, Craft N, Groshen S (2011). A critical role for GRP78/BiP in the tumor microenvironment for neovascularization during tumor growth and metastasis. Cancer Res.

[19] Zhang X, Zhang L, Wang S, Wu D, Yang W (2015). Decreased functional expression of Grp78 and Grp94 inhibits proliferation and attenuates apoptosis in a human gastric cancer cell line in vitro. Oncol Lett.

[20] Zhang LJ, Chen S, Wu P, Hu CS, Thorne RF, Luo CM, Hersey P, Zhang XD (2009). Inhibition of MEK blocks GRP78 up-regulation and enhances apoptosis induced by ER stress in gastric cancer cells. Cancer Lett.

[21] Winder T, Bohanes P, Zhang W, Yang D, Power DG, Ning Y, Gerger A, Wilson PM, Tang LH, Shah M, Lee AS, Lenz HJ (2011). GRP78 promoter polymorphism rs391957 as potential predictor for clinical outcome in gastric and colorectal cancer patients. Ann Oncol.

[22] Zheng HC, Takahashi H, Li XH, Hara T, Masuda S, Guan YF, Takano Y (2008). Overexpression of GRP78 and GRP94 are markers for aggressive behavior and poor prognosis in gastric carcinomas. Hum Pathol.

[23] Li LN, Wang CL, Cai M (2014). Expression of GRP78 in gastric adenocarcinoma and its relationship with cell proliferation and microvessel density. Chin J Gastroenterol Hepatol.

[24] Peng CL, Yang SY, Ji JF, Xu WW, Ji CF, Wang JH, Tan QH, Yang L (2013). The expression and clinical significance of GRP78 AND pERK in gastric adenocarcinoma, chronic atrophic gastritis and superficial gastritis. China Oncol.

[25] Yang L, Yang S, Liu J, Wang X, Ji J, Cao Y, Lu K, Wang J, Gao Y (2014). Expression of GRP78 predicts taxane-based therapeutic resistance and recurrence of human gastric cancer. Exp Mol Pathol.

[26] Zhang XC, Yang WL, Xu HF, Wu DQ, Zhang WF, Zhang DW (2005). Expression of endoplasmic reticulum molecular chaperone Grp78 in human gastric cancer tissues and its clinical significance. Chin J Exp Surg.

[27] Xu FY, Li N, Cai J, Zhang YM, Tang B, Niu YY, Peng ZH, Chen WS (2010). Expressions of GRP78 and KAI1 in human gastric carcinoma tissue and their clinical significance. Acta Acad Med Milt Tert.

[28] Zhang J, Jiang Y, Jia Z, Li Q, Gong W, Wang L, Wei D, Yao J, Fang S, Xie K (2006). Association of elevated GRP78 expression with increased lymph node metastasis and poor prognosis in patients with gastric cancer. Clin Exp Metastasis.

[29] Fu ZQ, Zhen HY, Liu LJ (2014). Central role of GRP78 in growth of gastric carcinoma cells. Chin J Pathophysiol.

[30] Wei H, Jing H, Jiang Wu, Zhang DH (2014). Clinical importance of glucose-regulated protein 78 and glucose-regulated protein 94 expression ant its correlation with prognosis in gastric cancer. J Cap Med Univ.

[31] He ZX, Tang HL, Chen JF, Chen WL, Jiang WZ, Xie YH (2015). The expression and clinical pathological significance of GRP78 in human gastric cancer tissues. Med Sci J Cent South Chin.

[32] Hu YW, Han W, Zhu R, Cao F, Ding HZ (2016). Expression of GRP78 and KIAA1234 in gastric cancer and their prognostic value. Chin J Clin Oncol.

[33] Ge JJ, Yang SY, Chen HZ, Chen Y, Zhu J, Song JH, Yang L (2016). Expression of GRP78 and Ki-67 in gastric cancer and its relationship between clinical parameters. Med J Commun.

[34] Cook KL, Soto-Pantoja DR, Clarke PA, Cruz MI, Zwart A, Wärri A, Hilakivi-Clarke L, Roberts DD, Clarke R (2016). Endoplasmic reticulum stress protein GRP78 modulates lipid metabolism to control drug sensitivity and antitumor immunity in breast cancer. Cancer Res.

[35] Niu Z, Wang M, Zhou L, Yao L, Liao Q, Zhao Y (2015). Elevated GRP78 expression is associated with poor prognosis in patients with pancreatic cancer. Sci Rep.

[36] Zhao G, Kang J, Jiao K, Xu G, Yang L, Tang S, Zhang H, Wang Y, Nie Y, Wu K, Fan D, Zhang H, Zhang D (2015). High expression of GRP78 promotes invasion and metastases in patients with esophageal squamous cell carcinoma. Dig Dis Sci.

[37] Chang YJ, Chen WY, Huang CY, Liu HH, Wei PL (2015). Glucose-regulated protein 78 (GRP78) regulates colon cancer metastasis through EMT biomarkers and the NRF-2/HO-1 pathway. Tumour Biol.

[38] Zhang XX, Li HD, Zhao S, Zhao L, Song HJ, Wang G, Guo QJ, Luan ZD, Su RJ (2013). The cell surface GRP78 facilitates the invasion of hepatocellular carcinoma cells. Biomed Res Int.

[39] Xie J, Tao ZH, Zhao J, Li T, Wu ZH, Zhang JF, Zhang J, Hu XC (2016). Glucose regulated protein 78 (GRP78) inhibits apoptosis and attentinutes chemosensitivity of gemcitabine in breast cancer cell via AKT/mitochondrial apoptotic pathway. Biochem Biophys Res Commun.

[40] Li B, Cheng XL, Yang YP, Li ZQ (2013). GRP78 mediates radiation resistance of a stem cell-like subpopulation within the MCF-7 breast cancer cell line. Oncol Rep.

[41] Ciortea R, Malutan AM, Angheluta LM, Bucuri CE, Rada MP, Mihu D (2016). GRP78 levels, regional fat distribution and endometrial cáncer. Rev Med Chil.

[42] Raiter A, Vilkin A, Gingold R, Levi Z, Halpern M, Niv Y, Hardy B (2014). The presence of anti-GRP78 antibodies in the serum of patients with colorectal carcinoma: a potential biomarker for early cancer detection. Int J Biol Markers.

[43] Ren P, Chen C, Yue J, Zhang J, Yu Z (2017). High expression of glucose-regulated protein 78 (GRP78) is associated with metastasis and poor prognosis in patients with esophageal squamous cell carcinoma. Onco Targets Ther.

[44] Soudry E, Stern Shavit S, Hardy B, Morgenstern S, Hadar T, Feinmesser R (2017). Heat shock proteins HSP90, HSP70 and GRP78 expression in medullary thyroid carcinoma. Ann Diagn Pathol.

[45] Thornton M, Aslam MA, Tweedle EM, Ang C, Campbell F, Jackson R, Costello E, Rooney PS, Vlatković N, Boyd MT (2013). The unfolded protein response regulator GRP78 is a novel predictive biomarker in colorectal cancer. Int J Cancer.

[46] Fu W, Wu X, Li J, Mo Z, Yang Z, Huang W, Ding Q (2010). Upregulation of GRP78 in renal cell carcinoma and its significance. Urology.

[47] Zhang LH, Yang XL, Zhang X, Cheng JX, Zhang W (2011). Association of elevated GRP78 expression with increased astrocytoma malignancy via Akt and ERK pathways. Brain Res.

[48] Daneshmand S, Quek ML, Lin E, Lee C, Cote RJ, Hawes D, Cai J, Groshen S, Lieskovsky G, Skinner DG, Lee AS, Pinski J (2007). Glucose-regulated protein GRP78 is up-regulated in prostate cancer and correlates with recurrence and survival. Hum Pathol.

[49] Wang Q, He Z, Zhang J, Wang Y, Wang T, Tong S, Wang L, Wang S, Chen Y (2005). Overexpression of endoplasmic reticulum molecular chaperone GRP94 and GRP78 in human lung cancer tissues and its significance. Cancer Detect Prev.

[50] Shuda M, Kondoh N, Imazeki N, Tanaka K, Okada T, Mori K, Hada A, Arai M, Wakatsuki T, Matsubara O, Yamamoto N, Yamamoto M (2003). Activation of the ATF6, XBP1 and grp78 genes in human hepatocellular carcinoma: a possible involvement of the ER stress pathway in hepatocarcinogenesis. J Hepatol.

[51] Lee HY, Jung JH, Cho HM, Kim SH, Lee KM, Kim HJ, Lee JH, Shim BY (2015). GRP78 protein expression as prognostic values in neoadjuvant chemoradiotherapy and laparoscopic surgery for locally advanced rectal cancer. Cancer Res Treat.

[52] Shimizu A, Kaira K, Yasuda M, Asao T, Ishikawa O (2017). Clinical and pathological significance of ER stress marker (BiP/GRP78 and PERK) expression in malignant melanoma. Pathol Oncol Res.

[53] Kaira K, Toyoda M, Shimizu A, Imai H, Sakakura K, Nikkuni O, Suzuki M, Iijima M, Asao T, Chikamatsu K (2016). Decreasing expression of glucose-regulated protein GRP78/BiP as a significant prognostic predictor in patients with advanced laryngeal squamous cell carcinoma. Head Neck.

[54] Kaira K, Toyoda M, Shimizu A, Mori K, Shino M, Sakakura K, Takayasu Y, Takahashi K, Oyama T, Asao T, Chikamatsu K (2016). Expression of ER stress markers (GRP78/BiP and PERK) in patients with tongue cancer. Neoplasma.

[55] Ma X, Guo W, Yang S, Zhu X, Xiang J, Li H (2015). Serum GRP78 as a tumor marker and its prognostic significance in non-small cell lung cancers: A retrospective study. Dis Markers.

